# A Prospective Study on the Association Between Grip Strength and Cognitive Function Among Middle-Aged and Elderly Chinese Participants

**DOI:** 10.3389/fnagi.2019.00250

**Published:** 2019-09-10

**Authors:** Yong Liu, Xinyi Cao, Nannan Gu, Bixi Yang, Jijun Wang, Chunbo Li

**Affiliations:** ^1^Shanghai Key Laboratory of Psychotic Disorders, Shanghai Mental Health Center, Shanghai Jiao Tong University School of Medicine, Shanghai, China; ^2^Clinical Neurocognitive Research Center, Shanghai Mental Health Center, Shanghai Jiao Tong University School of Medicine, Shanghai, China; ^3^Center for Excellence in Brain Science and Intelligence Technology, Chinese Academy of Sciences, Shanghai, China; ^4^Institute of Psychology and Behavioral Science, Shanghai Jiao Tong University, Shanghai, China; ^5^Brain Science and Technology Research Center, Shanghai Jiao Tong University, Shanghai, China

**Keywords:** aging, cognitive function, grip strength, prospective study, predictor

## Abstract

**Objective:**

To study the efficacy of grip strength (GS) as a predictor of cognitive function in a large, nationwide sample of Chinese participants aged 45 years and above.

**Methods:**

We used data from three waves (W1, W2, and W3) fielded by the China Health and Retirement Longitudinal Study (CHARLS). Cognitive function was tested biennially and calculated using two categories: episodic memory and mental intactness. Demographics, health behaviors, and medical conditions were considered potential confounders. Using multivariate linear regression models (MLRMs), we examined the association between baseline GS (measure in W1) and cognitive function in W3. Using a generalized estimating equation (GEE), we examined baseline GS as a predictor of cognitive function change.

**Results:**

Total 9,333 individuals (53.2% women), with a mean baseline episodic memory score of 6.5, mean baseline mental intactness score of 7.2, and aged over 45 years (mean age = 58.6), were selected. The mean follow-up time was 4.0 years (range: 3.3–5.0 years). Using MLRMs and comparing the lowest GS score with the highest baseline GS score, we observed a significant correlation with a higher global cognitive function in both women (β = 1.061, *p* < 0.001) and men (β = 1.233, *p* < 0.001). After adjusting baseline global cognition, the highest GS level was still statistically significant in both women (β = 0.543, *p* < 0.05) and men (β = 0.742, *p* < 0.001). GEE suggested that the participants in the highest GS quartile had better cognitive performance over time in both women (β = 0.116, *p* = 0.030) and men (β = 0.143, *p* = 0.008) than those in the lowest quartile.

**Conclusion:**

Higher baseline level of GS was significantly related to better cognitive function and slowed the rate of its decline. Thus, it is an independent predictor of better cognitive status in middle-aged and elderly Chinese.

## Introduction

Cognitive disorders (CDs), also known as neurocognitive disorders (NCDs), are a category of mental health disorders that primarily affect cognitive abilities such as learning, memory, perception, and problem solving. NCDs include delirium and mild to major NCD (previously known as dementia) (Simpson, 2014), which contribute to the disability and decreased life-span, considerably affecting quality of life in the elderly (Murray et al., 2013). Currently there are no cures for these diseases, thus, identifying predictive clinical signs of cognitive decline and dementia is imperative for the implementation of an adapted care. However, the complex association between physical performance and cognitive function might provide an insight into the possible therapeutic and prophylactic measures in these diseases (Amieva et al., 2005). Previously, grip strength (GS) has been represented as a predictive factor for Alzheimer’s disease (AD) (Rijk et al., 2015), considering that cognitive impairments, AD and other common neurodegenerative diseases, are preceded by a “silent” clinical period that can last longer than a decade. Identifying such “soft” physical signs associated with the progressive decline of cognitive function has important implications in the early intervention for these illnesses.

Several studies aimed to assess the associations between GS and cognitive decline or dementia; some (Camargo et al., 2016; Praetorius Björk et al., 2016; Veronese et al., 2016; Hooghiemstra et al., 2017), but not all (Atkinson et al., 2010), reported a positive relationship. It is evident that poor GS is associated with a greater risk of dementia. Furthermore, a small number of studies have suggested that higher GS at baseline is a protective factor in preventing the development of AD (Rijk et al., 2015). However, their cross-sectional and longitudinal association have not been fully investigated, and thus, remain unclear in China. Therefore, we aimed to examine the predictive accuracy of baseline GS levels for cognitive function as well as its slow decline over time in a large, population-based sample derived from the “China Health and Retirement Longitudinal Study (CHARLS).”

## Materials and Methods

### Study Sample

**FIGURE 1:**
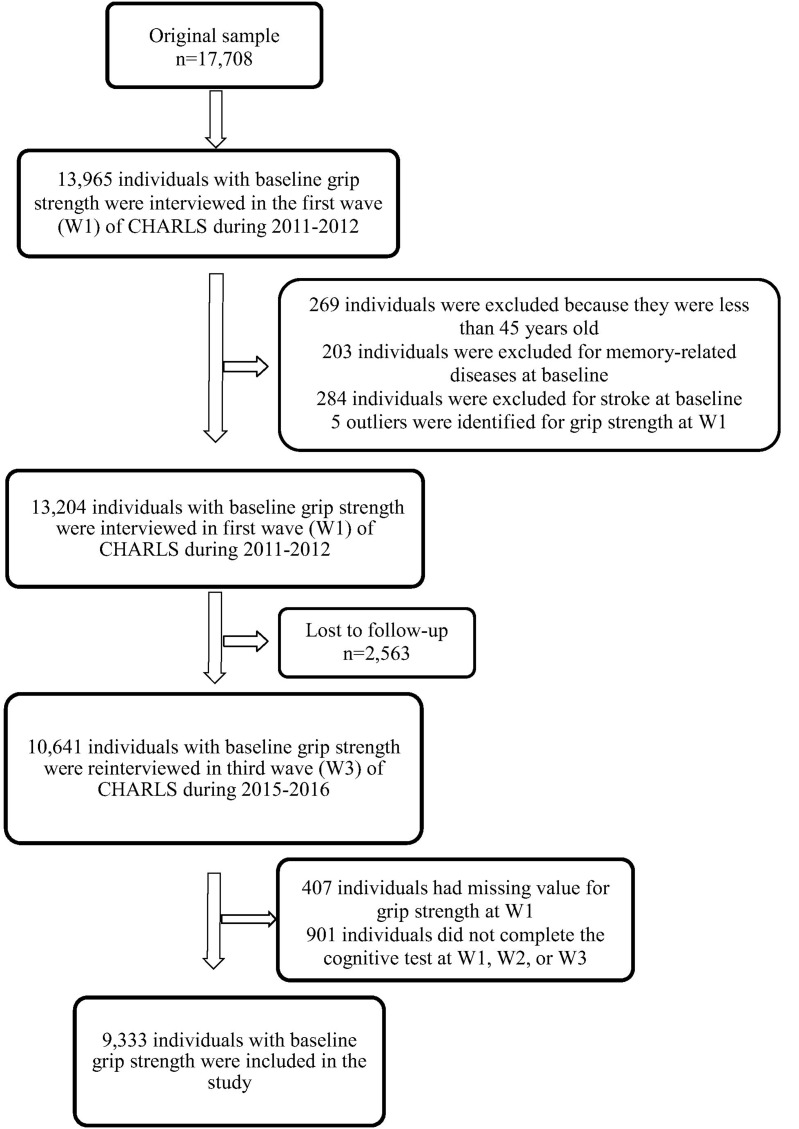
Study diagram.

The China Health and Retirement Longitudinal Study is a nationwide longitudinal survey conducted by the National School of Development at Peking University in China on people above 45 years of age. The data of CHARLS is publicly accessible. Researchers could apply for the data by signing a data usage agreement online and providing his/her basic information. Details of the survey protocol and implementation involved in the CHARLS have previously been described (Zhao et al., 2014). The survey included three waves covering 150 county-level units distributed in 28 provinces of China. The baseline (W1) survey was conducted in 2011–2012 on 17,708 participants with a high response rate. But only 78.9% of them did physical performance measures (Zhao et al., 2014), reducing the sample to 13,965 individuals. Compared to the baseline sample (*n* = 17,705), this subsample were, on average, significantly older (*p* = 0.006), with a higher proportion of females (*p* = 0.043), people with married status (*p* < 0.001) and people less educated (*p* < 0.001) (Supplementary Table S1). Of the 13,965 individuals, 13,204 individuals with baseline GS was included (269 individuals were excluded because they were less than 45 years old, 203 individuals were excluded for memory-related diseases at baseline, 284 individuals were excluded for stroke history at baseline and 5 outliers were identified for GS at W1). The third wave (W3) survey successfully re-interviewed 10,641 of these individuals in 2015–2016, and 2,563 (19.4%) were lost to follow-up. All surveys, including the questionnaire, laboratory measurements, and physical function were administered by well-trained clinicians in a face-to-face setting. Here, 9,333 individuals who underwent the three wave surveys were included (407 individuals had missing value for GS at W1, 901 individuals did not complete the cognitive test at W1, W2, or W3). There were no significant demographic characteristics (gender and educational attainment), heath status (other than hearing problems), health behavior differences between the baseline participants 13,965 and the third wave 9,333. Compared to the baseline sample, 9,333 individuals were significantly younger (*p* < 0.001), with higher proportion of married status (*p* < 0.001), a lower proportion of hearing problems (*p* < 0.001) and better average cognitive (*p* < 0.001) and GS (*p* < 0.001) scores (Table 1). Study diagram and exclusion criteria were listed in the Figure 1.

**TABLE 1:** Demographic characteristics of the samples.

	**Wave 3 (*N* = 9,333)**	**Baseline sample (*N* = 13,965)**	***p*-value**
Follow-up time(years), mean ± SD	4.0 ± 0.1(3.3–5.0)		
Age(years), mean	58.6 ± 8.7	59.3 ± 10.0	**<0.001**
**Gender (%)**			0.920
Male	4365(46.8)	6522(46.7)	
Female	4968(53.2)	7443(53.3)	
Marital status (married) (%)	7908(84.7)	11488(82.3)	**<0.001**
**Educational attainment (%)**			0.946
≤primary school	6456(69.2)	9666(69.2)	
>primary school	2877(30.8)	4299(30.8)	
**Baseline cognition, mean ± SD**			
Global cognition	13.7 ± 5.7	13.1 ± 6.2	**<0.001**
Episodic memory	6.5 ± 3.7	6.2 ± 3.9	**<0.001**
Mental intactness	7.2 ± 3.1	6.9 ± 3.3	**0.003**
**Health status**			
Hypertension (%)	2116(22.7)	3370(24.1)	0.010
Fall-related injuries (%)	1471(15.8)	2241(16.1)	0.559
Hip fracture (%)	146(1.6)	224(1.6)	0.812
Dyslipidemia (%)	794(8.5)	1241(8.9)	0.315
Diabetes or high blood sugar (%)	498(5.3)	796(5.7)	0.248
Cancer or malignant tumor (%)	83(0.9)	141(1.0)	0.356
Heart problems (%)	1023(11.0)	1629(11.7)	0.097
Near-vision impairment (%)	2178(23.3)	3218(23.0)	0.603
Far-vision impairment (%)	2015(21.6)	3107(22.3)	0.234
Hearing problems (%)	1168(12.5)	2067(14.8)	**<0.001**
Depressive symptoms (CES-D), mean ± SD	19.7 ± 4.9	19.5 ± 5.6	0.627
**Health behaviors**			
Smoking (%)	3633(38.9)	5481(39.3)	0.622
Drinking (%)	2356(25.2)	3406(24.4)	0.139
**Body mass index (kg/m^2^)**			0.056
Thin(<18.5)	581(6.2)	964(6.9)	
Normal(18.5–24)	4938(52.9)	7448(53.3)	
Overweight(≥24)	3814(40.8)	5553(39.8)	
Grip strength (kg), mean ± SD	33.0 ± 10.2	32.3 ± 10.5	**<0.001**
Male	39.7 ± 8.9	38.9 ± 9.46	
Female	27.1 ± 7.3	26.6 ± 7.6	
**Grip strength (kg) quartiles**			
*Male, n (%), mean ± SD*			
Q1(≤34 kg)	1140(26.6), 28.8 ± 4.8		
Q2(34–40 kg)	1250(28.7), 37.5 ± 1.9		
Q3(40–45.2 kg)	880(19.7), 42.9 ± 1.5		
Q4(>45.2 kg)	1095(25.0), 51.0 ± 4.6		
*Female, n (%), mean ± SD*			
Q1(≤22.5 kg)	1281(25.1), 18.3 ± 3.7		
Q2(22.5–27 kg)	1282(26.9), 25.1 ± 1.2		
Q3(27–31 kg)	1173(23.5), 29.3 ± 1.1		
Q4(>31 kg)	1232(24.5), 36.2 ± 4.9		

*SD, standard deviation.*

### Cognitive Function

Cognitive performance, in CHARLS, was calculated using two categories: episodic memory and mental intactness. Each respondent was asked to immediately repeat as many Chinese nouns as possible, from a list read to him/her (immediate word recall) and to recall the same 5 min later (delayed recall) (Wang et al., 2017). Episodic memory was defined as the summation of immediate and delayed recall scores ranging from 0 to 20. Mental intactness scores, which included numerical ability, time orientation, and picture drawing, were obtained from the following set of questions: serial sevens, temporal orientation (date, month, year, day of week, and season), and intersecting pentagon copying test. Answers to these questions were accumulated into a score named mental intactness ranging from 0 to 11. The global cognitive function was the summation of the episodic memory and mental intactness scores. Baseline cognition scores were calculated at W1.

### Grip Strength

Grip strength (kilogram) was estimated through the dynamometer (WCS-100, Nantong, China). Individuals needed to squeeze the handles as long and as tightly as possible or until the needle stopped rising. Individuals also needed to be in a standing position with their arms hanging naturally at their sides. Additional measurements were recorded for each hand, while alternating the sides, giving a total of two readings for each side. The best of the four GS measurements was used in statistical analyses. We conducted the analysis separately for men and women to identify gender differences in muscle strength (Metter et al., 1997; Gallagher and Heymsfield, 1998; Baumgartner et al., 1999). GS scores were divided into quartiles, independently, for both the sexes. We categorized the GS scores of ≤34.0 kg, 34.0–40.0 kg, 40.0–45.2 kg, and >45.2 kg as Q1, Q2, Q3, and Q4, respectively, for men. Similarly, for women GS scores of ≤22.5 kg, 22.5–27.0 kg, 27.0–31.0 kg, and >31.0 kg were categorized as Q1, Q2, Q3, and Q4, respectively.

### Potential Confounders

We also included other covariates such as the following: age, follow-up time, educational attainment, smoking, drinking, body mass index (BMI), hypertension, fall-related injuries, hip fracture, dyslipidemia, diabetes or high blood sugar, cancer or malignant tumor, heart problems, stroke, near and far-vision impairment, hearing problems, memory-related diseases, and depressive symptoms. Educational attainment was categorized as either “lower” or “higher” than primary school. Smoking and drinking habits were classified as either “never” or “current.” Depressive symptoms were assessed using the 10-item Center for Epidemiologic Studies Short Depression Scale (CES-D 10). Others were dichotomized as either “no” or “yes.”

### Statistical Analysis

First, descriptive statistics were used to show the characteristics of the study sample. The *t*-test/Mann–Whitney *U* test and chi-square test were used for comparison of baseline characteristics between two samples. The linear correlations between baseline GS and cognitive function in W3 were estimated using multivariate linear regression models (MLRMs) with potential confounders. Generalized estimating equation (GEE) was used to examine the predictive capability of baseline GS for changes in cognitive function over a period of 4 years. MLRMs was used to analyze the cross-sectional association using two models. We adjusted for age, education, marital status, health status, health behaviors, and BMI in model 1. Model 2 was further adjusted as model 1 with baseline cognition. GEE was used to analyze longitudinal association using three models. In model 1, the analysis was adjusted for baseline global cognition, age, follow-up time, education, marital status, BMI. Model 2 was adjusted as model 1 with further adjustment for GS. Model 3 was adjusted as model 2 with further adjustment for health status, health behaviors, and BMI. We chose GEE because it extends the generalized linear model to allow further analysis of longitudinal data. Secondly, because parameter estimation in GEE models remained relatively stable, it allowed us greater flexibility in modeling the effects of time on our results (Zeger and Liang, 1986; Zeger et al., 1988). All data were analyzed using STATA version 13 (StataCorp LP, College Station, TX, United States). The level of significance was set at *p* < 0.05.

## Results

Table 1 presents the baseline characteristics of the sample. A total of 9,333 individuals (4,365 men and 4,968 women) were included in the current study after excluding those who did not complete the necessary measurements at W1 or W3 and who were under 45 years of age at W1 (Figure 1). The mean participant age was 58.6 years [standard deviation (SD) = 8.7 years], 53.2% of the participants were women, and 84.7% were married. With regards to educational attainment, 30.8% attended primary school or above. Near-vision impairment (23.3%), hypertension (22.7%), far-vision impairment (26.1%), fall-related injuries (15.8%), hearing problems (12.5%), and heart problems (11.0%) were the most common medical conditions. The mean of follow-up time was 4.0 years (SD = 0.1 years), ranging from 3.3 to 5.0 years. Baseline GS ranged from 6 to 73 kg/m^2^ for men (mean = 39.7 kg/m^2^, SD = 8.9 kg/m^2^), and from 2 to 100 kg/m^2^ for women (mean = 27.1 kg/m^2^, SD = 7.3 kg/m^2^). The mean baseline global cognition score, episodic memory and mental intactness were 13.7 (SD = 5.7), 6.5 (SD = 3.7), and 7.2 (SD = 3.1), respectively.

Table 2 shows the relationship between the baseline GS level and baseline cognitive function through MLRMs. The higher GS significantly associated with better cognition in wave 1. After adjusting for potential confounders in female, referenced to the lowest GS level, the third quartile was the most highly associated with global cognition (β = 1.442, *p* < 0.001). Alternatively, for men, the highest GS level was the most highly related to global cognitive function (β = 1.388, *p* < 0.001).

**TABLE 2:** Association between baseline grip strength and baseline cognition by multivariate linear regression.

**Sex**	**Independent variable**	**Global cognition β(SE)**
Female	Q1(≤22.5 kg)	Ref.
	Q2(22.5–27 kg)	0.733(0.198)^∗∗∗^
	Q3(27–31 kg)	1.442(0.209)^∗∗∗^
	Q4(>31 kg)	1.239(0.216)^∗∗∗^
Male	Q1(≤34 kg)	Ref.
	Q2(34–40 kg)	0.900(0.194)^∗∗∗^
	Q3(40–45.2 kg)	1.155(0.221)^∗∗∗^
	Q4(>45.2 kg)	1.388(0.223)^∗∗∗^

*^∗∗∗^*p* < 0.001. Ref.: reference, β: beta coefficient, SE: standard error.*

Table 3 shows the relationship between the baseline GS level and the follow-up cognitive function in MLRMs. After adjusting for potential confounders in women, referenced to the lowest GS level, the third quartile was associated with better global cognition (β = 1.112, *p* < 0.001), and the highest GS level was associated with higher global cognitive function (β = 1.061, *p* < 0.001). In model 2, baseline global cognition was also included as an independent variable. The second quartile of GS level and the global cognitive function did not demonstrate statistically significant association. The third quartile was associated with better global cognition (β = 0.509, *p* < 0.05) and the highest GS level showed statistical significance (β = 0.543, *p* < 0.05). Alternatively, for men, the highest GS level was related to highest global cognitive function (β = 1.233, *p* < 0.001). In model 2, the second and third quartiles level of GS did not show statistical significance. However, positive correlations between the highest GS level and better cognitive measures were observed (β = 0.742, *p* < 0.001). Supplementary Figures S1, S2 show the plotted estimated average global cognition in W3 across the baseline GS and their 95% confidence interval for both women and men before and after adjusting for baseline global cognition.

**TABLE 3:** Association between baseline grip strength and follow-up cognition by multivariate linear regression.

**Sex**	**Independent variable**	**Global cognition β(SE)**
Female	Model 1	
	Q1(≤22.5 kg)	Ref.
	Q2(22.5–27 kg)	0.628(0.193)^∗∗^
	Q3(27–31 kg)	1.112(0.204)^∗∗∗^
	Q4(>31 kg)	1.061(0.210)^∗∗∗^
	Model 2	
	Q1(≤22.5 kg)	Ref.
	Q2(22.5–27 kg)	0.321(0.174)
	Q3(27–31 kg)	0.509(0.185)^∗^
	Q4(>31 kg)	0.543(0.191)^∗^
Male	Model 1	
	Q1(≤34 kg)	Ref.
	Q2(34–40 kg)	0.735(0.193)^∗∗∗^
	Q3(40–45.2 kg)	0.809(0.220)^∗∗∗^
	Q4(>45.2 kg)	1.233(0.222)^∗∗∗^
	Model 2	
	Q1(≤34 kg)	Ref.
	Q2(34–40 kg)	0.417(0.181)
	Q3(40–45.2 kg)	0.400(0.206)
	Q4(>45.2 kg)	0.742(0.209)^∗∗∗^

*^∗^*p* < 0.05, ^∗∗^*p* < 0.005, ^∗∗∗^*p* < 0.001. Ref.: reference, β: beta coefficient, SE: standard error.*

**FIGURE 2:**
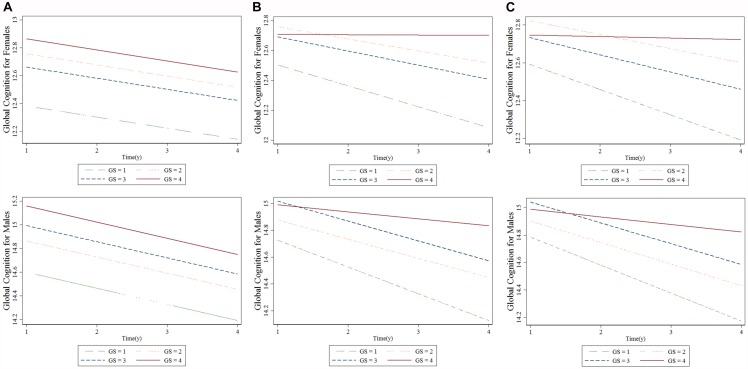
Longitudinal association between baseline grip strength (GS) and cognitive changes over time for females and males: generalized estimating equation. **(A)** Model 1, **(B)** Model 2, and **(C)** Model 3. “GS = 1” is the baseline GS first quartile, “GS = 2” is the baseline GS second quartile, “GS = 3” is the baseline GS third quartile, “GS = 4” is the baseline GS fourth quartile.

Table 4 summarizes the results from the GEE for GS quartiles as a predictor of cognition over a period of 4 years in a population of middle-aged and elderly individuals. The rate of decline in global cognition was 0.06 points every year. The fourth quartile of GS was associated with higher cognitive function in 4 years after adjusting for age, follow-up time, civil status, educational attainment, BMI, and baseline global cognition in model 1. The interaction between GS quartile and follow-up time (GS-by-time) was estimated in model 2. There were significant associations between individuals with the highest GS, indicating that people in highest GS quartile showed a significantly lower rate of decline in global cognition over time compared to those in the lowest quartile. The participants in the fourth quartile, compared to those in the first quartile, had a parameter estimate of 0.125 points per year (SE = 0.052; *p* < 0.05). In model 3, after adjusting for all covariates, the correlation between GS-by-time interaction (fourth quartile) and cognitive function over 4 years remained statistically significant (β = 0.116 with SE = 0.053, *p* = 0.030). Similar results (β = 0.143 with SE = 0.054, *p* = 0.008) were also observed for participants in fourth quartile compared to those in first quartile with regards to men. Other factors, such as older age and hearing problems, were associated with a decline in global cognition score in women. However, factors such as far-vision impairment besides older age were associated with poor performance in global cognition for men. Higher educational attainment and marital status were associated with better cognitive function in both the sexes. Figure 2 depicted predictive margins from the GEE in women and men on different models, focusing on the variance in the predicted slopes.

**TABLE 4:** Longitudinal global cognition by baseline grip strength among middle-aged and elderly Chinese participants: generalized estimating equation (*N* = 9,333).

**Sex**	**Independent variable**	**Model 1 β(SE)**	**Model 2 β(SE)**	**Model 3 β(SE)**
Female	GS (kg) quartiles			
	Q1(≤22.3 kg)	Ref.	Ref.	Ref.
	Q2(22.3–27 kg)	0.342(0.104)^∗∗^	0.222(0.107)^∗^	0.201(0.110)
	Q3(27–31 kg)	0.256(0.111)^∗^	0.156(0.112)	0.116(0.114)
	Q4(>31 kg)	0.464(0.113)^∗∗∗^	0.087(0.115)	0.051(0.119)
	GS(kg) quartiles × time			
	Q1 × follow-up time		Ref.	Ref.
	Q2 × follow-up time		0.040(0.051)	0.038(0.052)
	Q3 × follow-up time		0.033(0.552)	0.030(0.054)
	Q4 × follow-up time		0.125(0.052)^∗^	0.116(0.053)^∗^
Male	GS (kg) quartiles			
	Q1(≤34 kg)	Ref.	Ref.	Ref.
	Q2(34–40 kg)	0.245(0.105)^∗^	0.096(0.120)	0.083(0.122)
	Q3(40–45 kg)	0.367(0.114)^∗∗^	0.226(0.131)^∗^	0.230(0.135)
	Q4(>45 kg)	0.553(0.116)^∗∗∗^	0.131(0.128)	0.075(0.131)
	GS (kg) quartiles × time			
	Q1 × follow-up time		Ref.	Ref.
	Q2 × follow-up time		0.050(0.055)	0.040(0.055)
	Q3 × follow-up time		0.035(0.058)	0.037(0.059)
	Q4 × follow-up time		0.140(0.053)^∗^	0.143(0.054)^∗^

*^∗^*p* < 0.05, ^∗∗^*p* < 0.005, ^∗∗∗^*p* < 0.001. Ref.: reference, β: beta coefficient, SE: standard error.*

## Discussion

We examined the longitudinal relationship between GS and cognitive performance in 9,333 middle-aged and elderly Chinese participants. The analysis identified that higher baseline GS level was associated with better cognitive function with ageing and lower rates of decline in cognitive performance over a period of 4 years in both men and women. Even after adjusting for the relevant, potentially confounding independent variables, the two parameters showed significant association.

Our findings are similar to those of previous studies, which demonstrated that GS could predict cognition over time (Stijntjes et al., 2016; Jeong and Kim, 2018; Wang et al., 2019), for instance, Veronese et al. (2016) found that lower handgrip strength could predict incident cognitive decline in a population of 1,249 elderly community dwellers over a period of 4.4 years. However, there were studies that reported results contrary to our findings, for example, a 6-year follow-up study by Atkinson et al. (2010) revealed that there was no significant association between physical performance (such as gait, balance, and GS) and cognitive changes in 1,793 elderly women. There are several reasons for the differences in the reported results, one of which could be that our study included a significantly larger sample size and demonstrated a better study design.

Mechanistically, our findings are in accordance with the most notable hypotheses known as the “common cause hypothesis,” which demonstrates that cognition and muscle strength may share the same brain regions and networks (Christensen et al., 2001). Several researchers have drawn similar conclusions by observing the association between gait and cognitive function (Demnitz et al., 2016; Kueper et al., 2017). Furthermore, they also introduced Motoric Cognitive Risk (MCR) syndrome based on these associations, which can be used to identify people at risk of dementia in the population (Ayers and Verghese, 2016). This form of bounded rationality provides a reasonably straightforward way to implement the concept that simple motor tests or physical functions could be studied as biomarkers for identifying patients at a higher risk of cognitive impairment and dementia. However, there is still no direct imaging evidence to prove the rationality of this theory. Although it could be speculated from some studies (Rosano and Snitz, 2018) that brain areas between motor coordination and cognitive function have an overlap, we would need a significantly intuitive research design to prove and refine this theory.

Recognition of early risk factors for CDs has paramount practical significance, particularly if the predictors were in the form of easily developed indicators. Training programs that improve balance and GS might also help to either prevent or slow cognitive decline in the elderly, particularly in those with reduced muscle strength. Lower grip strength, poor balance, and gait might be crucial identification markers for patients who require exercise programs. A number of randomized controlled trials reveal that exercise programs in elderly adults can improve both their physical and cognitive functions (Kim, 2011; Yoon et al., 2016). However, other studies show contradicting results (Emery and Gatz, 1990). This is an important area that requires further exploration.

One of the advantages of this prospective study was that it has a large number of subjects, hence drawing significantly reasonable conclusions. Last but not least, this study used a longitudinal design to confirm that GS predicts changes in cognition over a relatively long follow-up time in Chinese population. However, this study had several limitations. Firstly, the cognitive domains studied were relatively limited and we could not evaluate the specific cognitive domains. Secondly, 19.4% of the original participants were lost to follow-up and 12.3% of the re-interviewed subjects at W3 were not included in the study due to incomplete baseline GS test or incomplete cognitive test at W1, W2 or W3. Otherwise, their inclusion may have influenced the association between GS and cognitive function as determined in this study. Thirdly, other confounding factors, such as healthy diet and physical activity, may influence the results that could not be accounted for this time. The future study will include additional related factors, such as gait speed, balance and other physical measurements to verify the present conclusion.

## Conclusion

This study suggests that higher GS in middle-aged and elderly adults predicted better global cognition over 4 years, unaffected by confounding factors. We need further research to understand the possible underlying mechanisms that may affect muscle strength and cognitive decline. A better understanding of the association between muscle strength and cognition may help us in the early identification of age-related cognitive decline and in order to find participants who could benefit from training programs.

## Data Availability

The data used in this manuscript are from the China Health and Retirement Longitudinal Study (CHARLS). We applied the permission for the date access (http://charls.pku.edu.cn/zh-CN) and got the access to use it. Prof. Yaohui Zhao (National School of Development of Peking University), John Strauss (University of Southern California), and Gonghuan Yang (Chinese Center for Disease Control and Prevention) are the principle investigator of the CHARLS, and they make the data available online for academic use freely.

## Ethics Statement

Each participant included in this study signed a written informed consent form before taking the survey. Ethics approval for the data collection in the CHARLS was obtained from the Biomedical Ethics Review Committee of Peking University (IRB00001052-11015).

## Author Contributions

YL and CL designed the study. YL, XC, NG, and BY acquired the data. YL performed the statistical analysis, assisted by JW and CL. YL and CL drafted the manuscript. XC, NG, BY, and JW reviewed the manuscript. All authors approved the final version for submission.

## Conflict of Interest Statement

The authors declare that the research was conducted in the absence of any commercial or financial relationships that could be construed as a potential conflict of interest.
