# Supplementary material for: A Prospective Study on the Association Between Grip Strength and Cognitive Function Among Middle-Aged and Elderly Chinese Participants
Source: Front Aging Neurosci. 2019 Sep 10;11:250. doi: 10.3389/fnagi.2019.00250 (PMC6747049; doi:10.3389/fnagi.2019.00250)
Supplement: Supplementary file 1 [file Table_1.docx]

Supplementary Material

**Table S1. Baseline characteristics difference between the subjects with grip strength (n=13,965) and the baseline sample (N=17,705)**

| Characteristics | Study sample  (N=13,965) | Study sample  (N=17,705) | P value |
| --- | --- | --- | --- |
| Age(y), mean±SD^a^ | 59.3±10.0 | 59.1±10.2 | **0.006** |
| Gender (%) |  |  | **0.043** |
| Female | 7433(53.2) | 9221(52.1) |  |
| Marital status (married) (%) | 11488(82.3) | 14170(80.0) | **<0.001** |
| Educational level (%) |  |  | **<0.001** |
| ≤primary school | 9666(69.2) | 11755(66.4) |  |


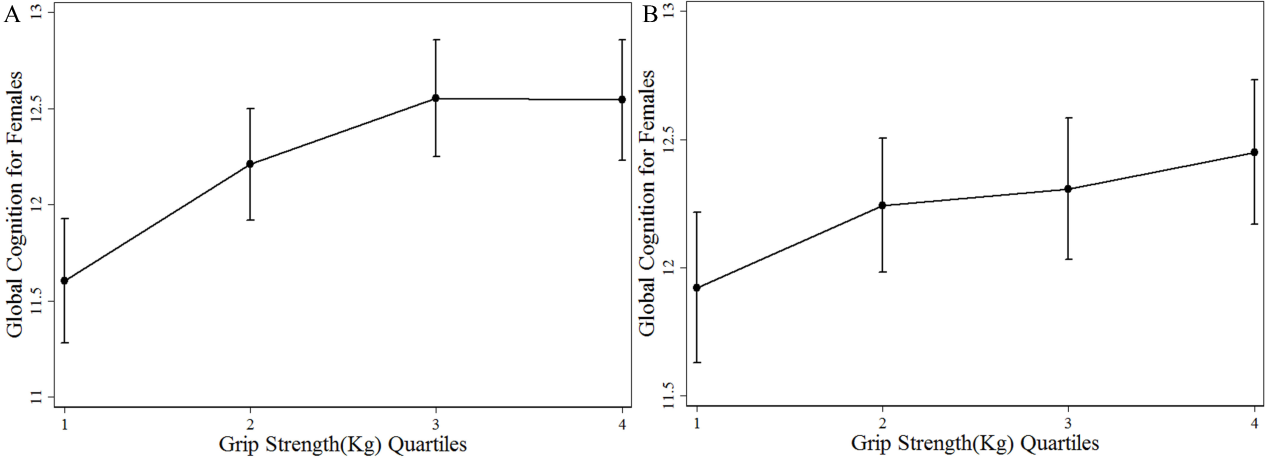


Figure S1. A and B show mean distributions of global cognition over a 4-year period using grip strength quartiles at baseline in women. A) shows data adjusted for age, education, marital status, health status, health behaviors, and body mass index. B) shows data adjusted for model 1+ baseline cognition.


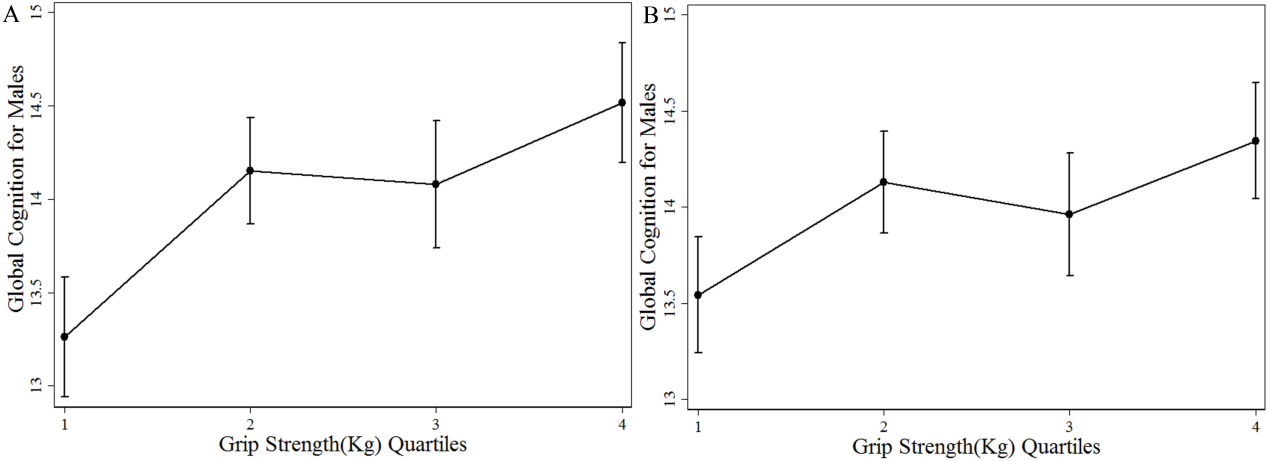


Figure S2. A and B show mean distributions of global cognition over a 4-year period using grip strength quartiles at baseline in men. A) shows data adjusted for age, education, marital status, health status, health behaviors, and body mass index. B) shows data adjusted for model 1+ baseline cognition.
